# Silver Nanoparticles Encapped by Dihydromyricetin: Optimization of Green Synthesis, Characterization, Toxicity, and Anti-MRSA Infection Activities for Zebrafish (*Danio rerio*)

**DOI:** 10.3390/ijms25105255

**Published:** 2024-05-11

**Authors:** Ling-Xiao Qi, Xue-Ting Wang, Jin-Ping Huang, Ting-Yan Yue, Yun-Shu Lu, Dong-Mei San, Yu-Xun Xu, Ya-Tong Han, Xiang-Yi Guo, Wei-Dong Xie, Yan-Xia Zhou

**Affiliations:** 1 Marine College, Shandong University, Weihai 264209, China; 202100700192@mail.sdu.edu.cn (L.-X.Q.); 202217720@mail.sdu.edu.cn (X.-T.W.); 202237725@mail.sdu.edu.cn (J.-P.H.); yuetingyan1026@163.com (T.-Y.Y.); 202100700190@mail.sdu.edu.cn (Y.-S.L.); 202000810109@mail.sdu.edu.cn (D.-M.S.); 202200700290@mail.sdu.edu.cn (Y.-X.X.); 202200700246@mail.sdu.edu.cn (Y.-T.H.); gxy5955@163.com (X.-Y.G.); 2 SDU-ANU Joint Science College, Shandong University, Weihai 264209, China

**Keywords:** DMY-AgNPs, optimization, MRSA, zebrafish

## Abstract

To achieve the environmentally friendly and rapid green synthesis of efficient and stable AgNPs for drug-resistant bacterial infection, this study optimized the green synthesis process of silver nanoparticles (AgNPs) using Dihydromyricetin (DMY). Then, we assessed the impact of AgNPs on zebrafish embryo development, as well as their therapeutic efficacy on zebrafish infected with *Methicillin-resistant Staphylococcus aureus* (MRSA). Transmission electron microscopy (TEM) and dynamic light-scattering (DLS) analyses revealed that AgNPs possessed an average size of 23.6 nm, a polymer dispersity index (PDI) of 0.197 ± 0.0196, and a zeta potential of −18.1 ± 1.18 mV. Compared to other published green synthesis products, the optimized DMY-AgNPs exhibited smaller sizes, narrower size distributions, and enhanced stability. Furthermore, the minimum concentration of DMY-AgNPs required to affect zebrafish hatching and survival was determined to be 25.0 μg/mL, indicating the low toxicity of DMY-AgNPs. Following a 5-day feeding regimen with DMY-AgNP-containing food, significant improvements were observed in the recovery of the gills, intestines, and livers in MRSA-infected zebrafish. These results suggested that optimized DMY-AgNPs hold promise for application in aquacultures and offer potential for further clinical use against drug-resistant bacteria.

## 1. Introduction

*Methicillin-resistant Staphylococcus aureus* (MRSA) infection afflicts approximately 119,000 individuals annually, resulting in nearly 20,000 deaths [1]. MRSA is as a predominant pathogen responsible for both nosocomial and community-acquired infections, posing a formidable threat to public health [2,3]. Current clinical treatments primarily rely on vancomycin, daptomycin, and linezolid to deal with MRSA infections [4]. However, the emergence of resistance to these antibiotics underscores the critical need for novel therapeutic strategies [5]. AgNPs have garnered extensive application in biomedical products, such as antiseptic dressings, textiles, and medicine delivery systems, owing to their remarkable antibacterial properties [6,7,8]. Furthermore, AgNPs are employed in aquacultures as antimicrobial agents, feed additives, and nano-vaccines [9]. Intriguingly, AgNPs present a low likelihood of microbial resistance development [10]. However, the widespread use and improper disposal of AgNPs inevitably contribute to their accumulation in aquatic systems [11]. Consequently, investigating the potential impacts of AgNPs on aquatic ecosystems has become increasingly imperative.

Traditional methods for the synthesis of AgNPs, such as chemical methods [12], photochemical methods [13], physical methods, etc., have high energy consumption, low conversion rates, and the use of toxic chemicals [14]. In contrast, green synthesis methods utilize cost-effective, efficient, and environmentally friendly reducing agents to rapidly synthesize stable AgNPs. In this study, we modified the green synthesis method of AgNP production using Dihydromyricetin (DMY). DMY is a natural dihydroxyflavone compound extracted from vine tea (*Ampelopsis grossedentata*) [15]. It is known for its multifaceted pharmacological properties, including antibacterial, anti-inflammatory, anticancer, antioxidant, and antidiabetic properties [16,17,18]. Flavonoids are prominent reducing agents in the green synthesis of AgNPs. While DMY has been utilized in this process, its resultant AgNPs exhibit larger particle sizes, scattered size distributions, and diminished stability [19].

The zebrafish (*Danio rerio*) is a tropical fish belonging to the super-class *Actinopterygii*, known for its advantages, such as short lifecycle and cost-effectiveness, and for its physiological similarity to many farmed species [20]. Notably, the zebrafish even exhibits remarkable genetic and physiological similarities to mammals, including humans [21]. The zebrafish embryo serves as a significant in vivo model for assessing the toxicity of nanomaterials due to its rapid embryonic development, ease of management, and visible embryologic phases [22]. Thus, zebrafish embryos and adult zebrafishes have been chosen in this work for use evaluating the in vivo toxicity and antibacterial efficacy of AgNPs, respectively.

This study presented an optimized process for synthesizing DMY-coated AgNPs, designed to address challenges such as large particle size, unclear DMY encapsulation, and low stability. Furthermore, the toxicity of AgNPs was thoroughly evaluated using zebrafish embryos in order to ascertain their environmental safety and suitability for application in aquaculture. Additionally, the therapeutic efficacy of AgNPs against drug-resistant bacterial infections was investigated using a model of adult zebrafish infected with MRSA. Our results revealed that high concentrations of AgNPs impacted the development of zebrafish embryos. Moreover, AgNPs demonstrated significant therapeutic effects on the intestines, gills, and livers of MRSA-infected zebrafishes.

## 2. Results and Discussion

### 2.1. Optimized Green Synthesis of DMY-AgNPs

**Figure 1:**
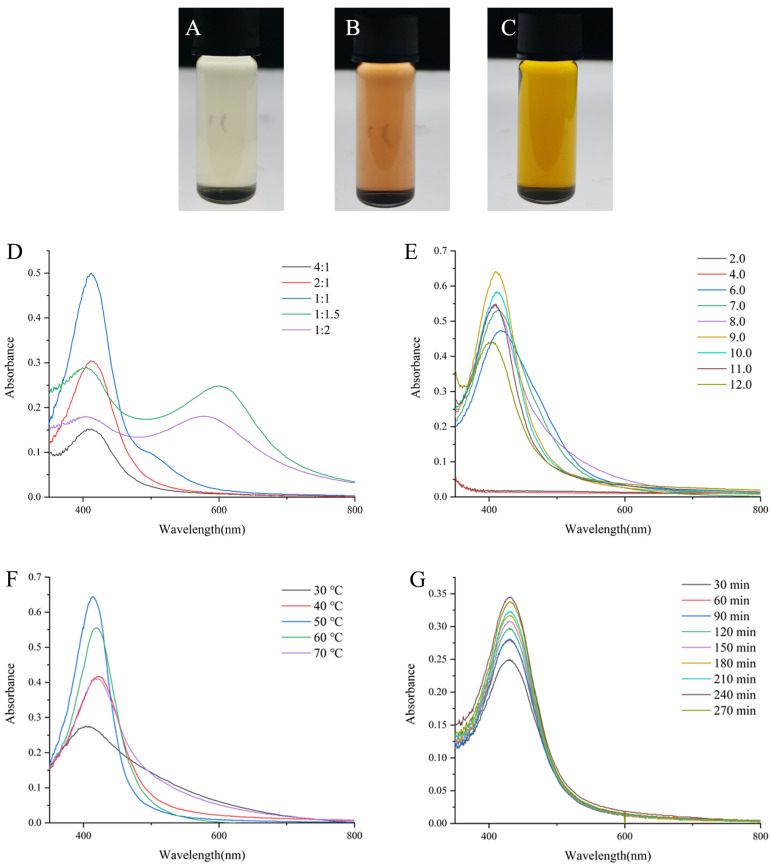
The lab photograph of (**A**) DMY solution, (**B**) DMY solution mixed with NaOH solution and (**C**) DMY-AgNPs (prepared under the optimal conditions). UV-vis spectra for DMY-AgNPs synthesized in different (**D**) DMY volume, (**E**) pH, (**F**) temperature and (**G**) time.

The formation of DMY-AgNPs was assessed through the changes in color and the UV–visible (UV-vis) spectrum. Occurring due to the excitation of surface plasmon resonance (SPR) in metal nanoparticles, a brown color represents the formation of AgNPs [23]. This color change was observed in the sample vials, confirming the synthesis of AgNPs (Figure 1A–C). Furthermore, a sharp peak was observed between 400 nm to 450 nm in the UV-vis spectra of AgNPs, consistent with previous findings [24]. In addition, the particle size and grain-size distribution of DMY-AgNPs were preliminarily analyzed using UV-vis spectroscopy. The scattering theory proposed by Mie G. [25] states that the peak value, wavelength, and width of the maximum absorption peak of AgNPs are related to their concentration, particle size, and grain size distribution, respectively.

Figure 1D illustrates a trend where the absorbance of AgNPs initially increased with the increasing DMY solution volume. However, this trend reversed at higher DMY concentrations, resulting in a subsequent decrease in absorbance. In the UV-vis spectra of the samples corresponding to the molar ratios of 1:1, 1:1.5 and 1:2, longitudinal plasmon band of AgNPs at 500–700 nm may be caused by the formation of non-spherical AgNPs [26]. Moreover, notable variations in the absorbance of AgNPs were observed under different pH conditions, with the strongest absorption SPR peak recorded at pH 9.0 (Figure 1E). This finding aligns with previous studies, such as that by Khalil, MA et al. [27]. It has been reported by Verma, A and Mehata, MS that an alkaline pH facilitates the deprotonation of hydroxyl groups in biomolecules, potentially influencing their capping and stabilizing capabilities [28]. To examine the influence of temperature on AgNPs synthesis, varying temperatures were used to obtain AgNPs. AgNPs prepared at 50 °C showed the highest SPR peak, and this gradually decreased as the temperature continually progressed (Figure 1F). As previously described in other studies, elevated temperatures expedite the reduction of silver ions, thereby enhancing the rate of AgNP formation [29]. However, excessively high temperatures may lead to aggregation, resulting in a decline in the maximum SPR absorbance peak [30]. Furthermore, the maximum absorption peak of AgNPs revealed significant variations, corresponding to the different reaction times. As illustrated in Figure 1G, the production of AgNPs increased with reaction times. Beyond 240 min, there was no significant change in the maximum SPR absorbance peak, indicating the completion of the reaction. In brief, the optimal synthesizing conditions for DMY-AgNPs were identified using a DMY solution with a pH of 9.0 in a 1:1 molar ratio (10 mM DMY to 10 mM AgNO_3_) at 50 °C for a duration of 240 min. The synthesized DMY-AgNPs solution had a pH value of 5.9.

### 2.2. Characterization of Silver Nanoparticles

#### 2.2.1. TEM and DLS Analysis

**Figure 2:**
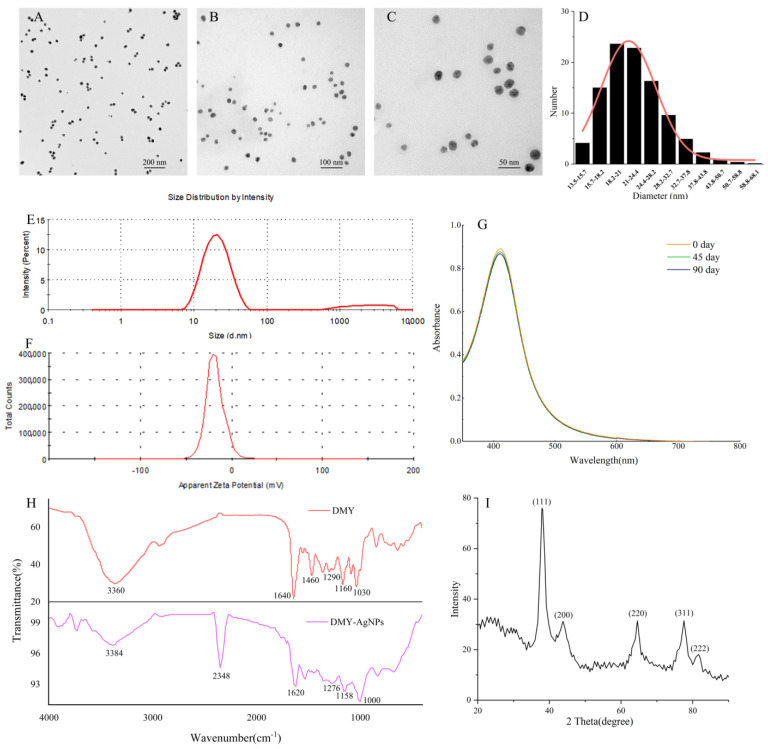
(**A**–**C**) TEM images of DMY-AgNPs. (**D**) The size distribution histogram of DMY-AgNPs. (**E**) The size of DMY-AgNPs. (**F**) Zeta potential values of DMY-AgNPs. (**G**) The storage stability of DMY-AgNPs. (**H**) FTIR spectra for DMY and DMY-AgNPs. (**I**) XRD pattern of DMY-AgNPs.

To study the morphological properties of DMY-AgNPs, including size, shape, and dispersion, the TEM images were analyzed (Figure 2A–C). The results revealed that DMY-AgNPs exhibited primarily spherical shapes, with an average size of 23.6 nm (Figure 2D). Moreover, the transparent cladding layer on the surface of DMY-AgNPs intuitively indicated the capping and encapsulation of DMY, contributing to the stability and dispersion of the AgNPs. DLS analysis was instrumental in assessing the average particle size and size distribution of the nanoparticles. As shown in Figure 2E, the average size of DMY-AgNPs was 19.9 ± 1.17 nm, with a PDI of 0.197 ± 0.0196. These findings indicate the superior properties of small particle size, narrow size distribution, and excellent polydispersity, corroborating the finding of the TEM analysis. The zeta potential value was used to determine the surface charge and stability of DMY-AgNPs in aqueous solutions. As indicated in Figure 2F, the zeta potential of DMY-AgNPs was negative (−18.1 ± 1.18 mV), from which it could reasonably be assumed that DMY was involved in both the synthesis and coating of AgNPs, contributing to their excellent stability [31]. We simultaneously investigated the storage stability of DMY-AgNPs over a period of 90 days. The UV-vis spectra remained consistent, without showing significant changes, which further affirmed the excellent stability of DMY-AgNPs (Figure 2G). Interestingly, DMY-AgNPs synthesized through optimization exhibited smaller particle sizes, more concentrated particle size distributions, and greater stability compared to previously reported DMY-AgNPs, which had particle sizes of 114.76 ± 1.34 nm, a PDI value of 0.301, and a zeta potential value of −16.5 ± 2.1 mV.

#### 2.2.2. FTIR Analysis

Fourier transform infrared spectroscopy (FTIR) was performed to study the interaction between DMY and AgNPs. According to the FTIR spectrum obtained (Figure 2H), DMY had a broad band at 3360 cm^−1^, which was attributed to the stretching vibration of N-H and O-H groups [32,33]. The peaks at 1640 cm^−1^ and 1460 cm^−1^ were attributed to the bending vibrations of -C=O and C-OH, respectively [34]. The peak at 1160 cm^−1^ was due to the skeletal C-O and C-C stretching vibrations in glycosidic linkage [35]. The development of -OH bends due to phenols or tertiary alcohols of flavonoids was evidenced by the peaks at 1290 cm^−1^ and 1030 cm^−1^ [36]. The characteristic peaks of DMY were still found in the FTIR spectra of DMY-AgNPs. Compared with DMY, a new peak was found at 2348 cm^−1^ (C-H), indicating that AgNO_3_ interacted with DMY [37].

#### 2.2.3. XRD Analysis

The crystalline structure of DMY-AgNPs was determined via X-ray diffraction (XRD), with the result shown in Figure 2I. The sharp XRD peaks at 37.79°(111), 43.62°(200), 64.43°(220), 77.39°(311), and 81.38°(222) were consistent with the standard data regarding face-centered cubic Ag (JCPDS No. 01-1164). The findings aligned well with numerous other reports pertaining to the structure of AgNPs, thus providing further confirmation of the successful formation of AgNPs.

### 2.3. Antibacterial Activity

**Figure 3:**
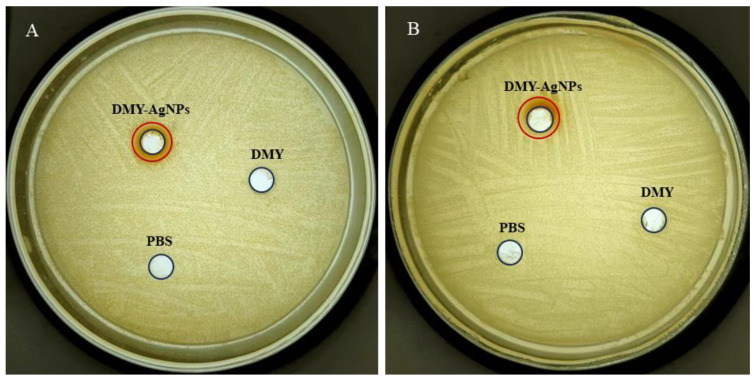
Circle of inhibition tests for DMY-AgNPs in (**A**) *S. aureus* and (**B**) MRSA.

We assessed the antibacterial activity of DMY-AgNPs against *Staphylococcus aureus* (*S. aureus*) and MRSA. According to the SNV 1959-1992 standard antimicrobial test, inhibition zones larger than 1 mm are considered indicative of antimicrobial activity [38]. As shown in Figure 3, compared with the PBS and DMY solution, DMY-AgNPs, respectively, exhibited clear inhibition zones of 1.92 mm and 1.75 mm for *S. aureus* and MRSA, demonstrating potent antibacterial activity. Furthermore, the minimal inhibitory concentrations (MICs) of DMY-AgNPs against *S. aureus* and MRSA were 0.781 and 1.56 μg/mL, respectively, while the corresponding minimum bactericidal concentration (MBC) values were 12.5 and 25.0 μg/mL. These results indicated that the antibacterial efficacy of DMY-AgNPs surpassed that of other green-synthesized AgNPs [39]. Previous studies have indicated that AgNPs adhere to the surface of cell membranes to penetrate into the cell, subsequently influencing the shape and function of the cell membranes by gathering along the metabolic pathway [40]. Thereafter, AgNPs interact with the basic components of bacterial cells and induce cell lysis and leakage, ultimately leading to cell death [41]. Smaller particles can facilitate the aforementioned process, contributing to their superior bactericidal capability [42]. Moreover, AgNPs exhibit bactericidal effects by enhancing cell wall penetration and inducing reactive oxygen species (ROS)-mediated peroxidation damage to DNA, proteins, and other intracellular components. This mechanism effectively circumvents MRSA’s inherent resistance mechanisms, including resistance genes such as the mecA gene, fem gene, and vanA gene, as well as β-lactamase and efflux activities. Consequently, MRSA demonstrates susceptibility to AgNPs, displaying similar MICs and MBCs to *S. aureus* [43].

### 2.4. Toxicity Test

**Figure 4:**
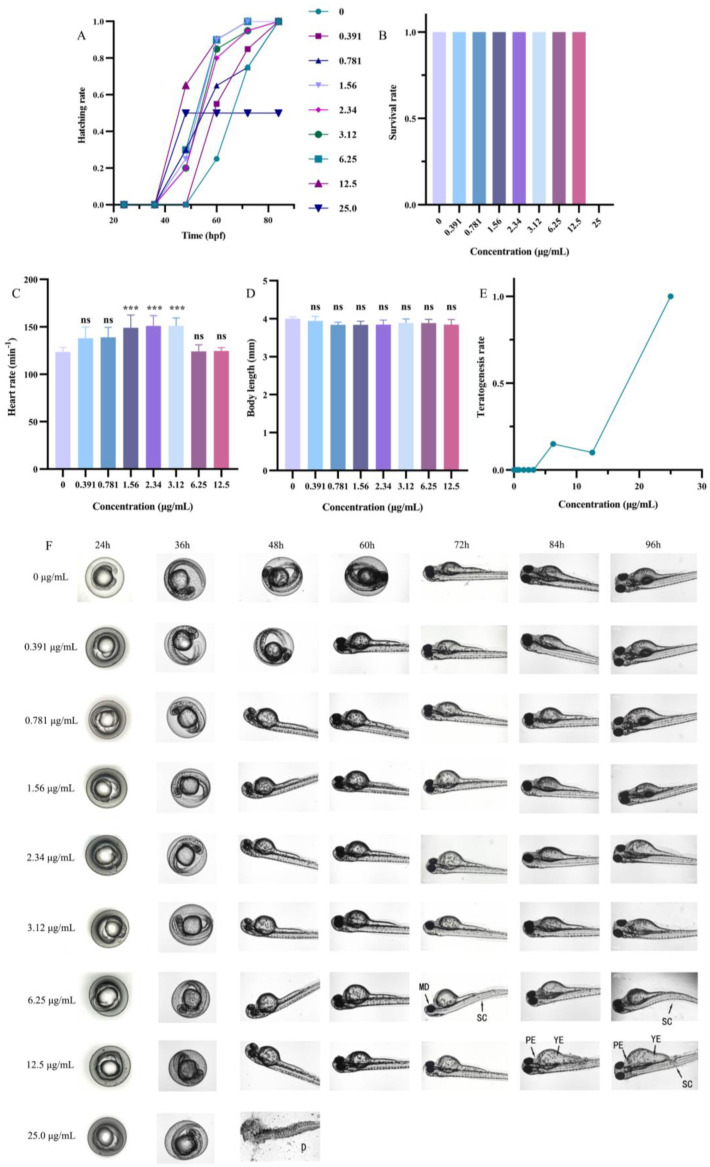
(**A**) Changes in hatching rate of zebrafish embryos exposed to DMY-AgNPs. Changes in (**B**) survival rate, (**C**) heart rate, (**D**) body length, and (**E**) teratogenesis of 96 hpf zebrafish embryos exposed to DMY-AgNPs (n = 6. *** *p* < 0.001. ‘ns’ means no significant difference). (**F**) Developmental status of zebrafish in different periods.

To explore the development effects of DMY-AgNPs on zebrafish embryos, we examined parameters including hatching rate, survival rate, heart rate, body length, and teratogenesis for different concentrations of DMY-AgNPs. As shown in Figure 4A,B, the hatchabilities and survival rates of zebrafish embryos were 100% with less than 12.5 µg/mL of DMY-AgNP treatment. However, at the higher exposure concentration (25.0 µg/mL), the hatchability and survival rate were recorded as being 50% and 0%, respectively. Previous studies have indicated that the toxicity of AgNPs to zebrafish embryos is influenced by various factors such as concentration, size, capping agents, and stability [44]. Remarkably, the DMY coating significantly mitigated the toxicity of AgNPs compared to other green-synthesized AgNPs with similar particle sizes [44]. Additionally, we also observed that low doses of DMY-AgNPs promoted the hatching of zebrafish embryos. Biplab Sarkar et al. also observed this phenomenon, demonstrating that AgNPs have a dual role of proliferation and anti-proliferation [45]. Furthermore, we observed the development of tachycardia in embryos exposed to 1.43 µg/mL, 2.15 µg/mL, 2.87 µg/mL, and 4.30 µg/mL of DMY-AgNPs (Figure 4C), which was potentially caused by a compensatory response to the circulatory disturbances induced by AgNPs [46]. Subsequent analysis revealed that the body lengths of zebrafish embryos exposed to various concentrations of DMY-AgNPs exhibited no significant variation (Figure 4D). When the concentration of DMY-AgNPs reached 6.25 and 12.5 µg/mL, only 15% and 10% of zebrafish exhibited abnormalities, respectively, including scoliosis (SC), pericardial edema (PE), yolk edema (YE), and mandibular deformity (MD) (Figure 4E,F) [47]. However, zebrafish began to die (D) at 25.0 µg/mL. The results indicated that low concentrations of DMY-AgNPs had minimal impact on the development of zebrafish embryos. Additionally, to understand the systemic toxicity of nanosilver, the biodistribution and neurodevelopmental toxicity of AgNPs in adult zebrafish remain to be further investigated [48]. Furthermore, the long-term exposure of adult zebrafish to DMY-AgNPs and its effects on the epigenetic patterns and multiple generations cannot be overlooked [49,50,51]. Further research is necessary to investigate these influence factors.

### 2.5. Therapeutic Efficacy

**Figure 5:**
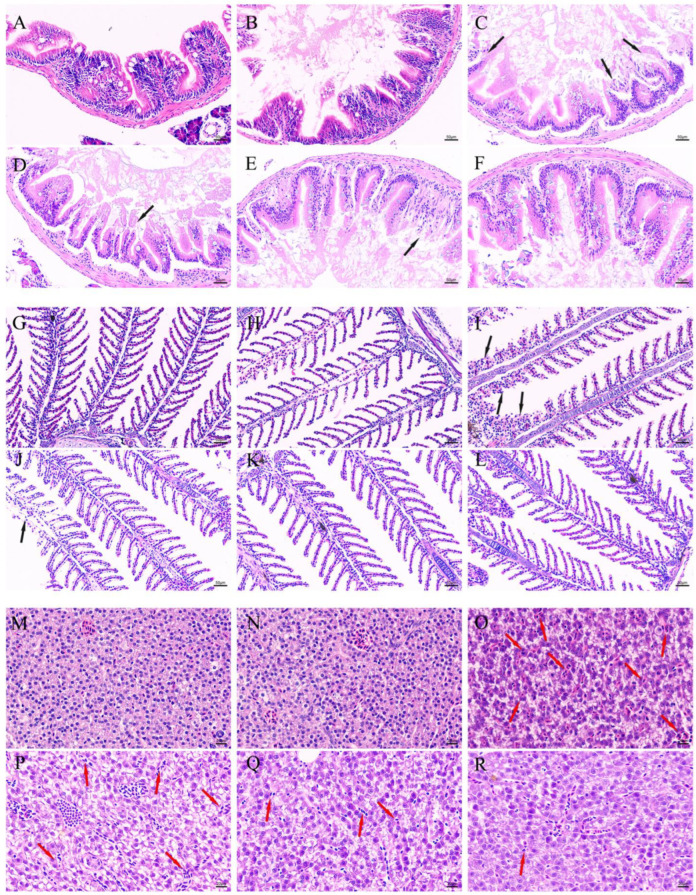
Intestines of (**A**) healthy, (**B**) wounded, (**C**) MRSA-infected, (**D**) 0.0156 μg/g DMY-AgNP-treated, (**E**) 0.0312 μg/g DMY-AgNP-treated, and (**F**) 0.0625 μg/g DMY-AgNP-treated adult zebrafish (black arrows represent cell shedding). Gills of (**G**) healthy, (**H**) wounded, (**I**) MRSA-infected, (**J**) 0.0156 μg/g DMY-AgNP-treated, (**K**) 0.0312 μg/g DMY-AgNP-treated, and (**L**) 0.0625 μg/g DMY-AgNP-treated adult zebrafish (black arrows represent loss of secondary lamellae). Livers of (**M**) healthy, (**N**) wounded, (**O**) MRSA-infected, (**P**) 0.0156 μg/g DMY-AgNP-treated, (**Q**) 0.0312 μg/g DMY-AgNP-treated, and (**R**) 0.0625 μg/g DMY-AgNP-treated adult zebrafish (red arrows represent congestion).

To assess the therapeutic efficacy of DMY-AgNPs in MRSA-infected zebrafish, histopathological examinations of intestines, gills, and livers tissues were conducted. The hematoxylin and eosin (HE) staining of colon tissue showed that in MRSA-infected groups, the villus structure was seriously damaged (black arrow). Conversely, the intestines of DMY-AgNP-treated zebrafish exhibited significant improvements, with reduced villus fracturing and shedding compared to the MRSA-infected group (Figure 5A–F). The histological examination of gills from MRSA-infected showed the grievous phenomenon of sub-epithelium cellular infiltration and the loss of secondary lamellae of epithelial cells (Figure 5G–L) (black arrow). However, in DMY-AgNP treatment group, the destruction of branchial epithelial tissue was alleviated a certain treatment dosage and this tissue recovered after the periodic feeding of 0.0625 μg/g of DMY-AgNPs. Compared to the control group, vacuolar degeneration and congestion (red arrow) were observed in the liver of MRSA-infected zebrafish (Figure 5O). The liver-related vacuolar degeneration and congestion of zebrafish were alleviated by treatment with DMY-AgNPs, and the degree of recovery was dose-dependent. (Figure 5P–R).

## 3. Materials and Methods

**Figure 6:**
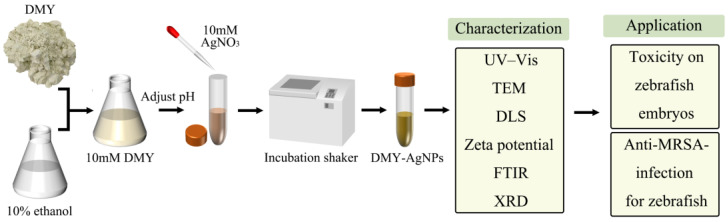
Schematic diagram of the green synthesis of AgNPs using DMY to ensure optimization, characterization, toxicity, and anti-MRSA-infection for zebrafish.

The method of green synthesis of AgNPs, using DMY to ensure optimization, characterization, toxicity, and anti-MRSA-infection for zebrafish, is illustrated in the schematic diagram (Figure 6).

### 3.1. Materials

Dihydromyricetin was purchased from Shanghai yuanye Bio-Technology Co., Ltd. (Shanghai, China). AgNO_3_ was obtained from Sinopharm Chemical Reagent Co., Ltd. (Shanghai, Chnia). H & E staining kit was purchased from Beijing Solarbio Science&Technology Co., Ltd. (Beijing, China). Methylene blue was purchased from Tianjin Hedong Hongyan Reagent Factory (Tianjin, China).

### 3.2. Optimized Process of DMY-AgNP Synthesis

We dissolved 320 mg of DMY in 100 mL of 10% ethanol. This filtered through a 10 μm filter membrane, and was added to 3 mL of 10 mM AgNO_3_ solution. This solution mixture was swayed and heated in an incubation shaker (QYC-2102, Shanghai Cimo Medical Instrument Co., Ltd., Shanghai, China) periodically (30, 60, 90, 120, 150, 180, 210, 240, and 270 min). The preparation parameter of AgNPs was optimized using single-factor experimentation, including the molar ratio of DMY to AgNO_3_ (4:1, 2:1, 1:1, 1:1.5, and 1:2), the pH (6.0, 7.0, 8.0, 9.0, 10.0, and 11.0), and the reaction temperatures (30, 40, 50, 60, and 70 °C).

### 3.3. Characterization of Silver Nanoparticles

The DMY-AgNPs solution was placed in a 2 mL sample vial for photograph observation. The absorbance spectra of DMY-AgNPs were determined using a UV-vis spectrophotometer (U-2910, Hitachi Ltd., Beijing, China)with a wavelength ranging from 350 nm to 800 nm. After drying off the DMY-AgNPs using the copper mesh, the TEM (JEM-1200EX, Japan Electron Optics Laboratory Co., Ltd., Beijing, China) was used to analyze the size and morphology of the DMY-AgNPs with an accelerating voltage of 100 kV. The particle size distribution, PDI, and zeta potential were analyzed using DLS (Nano ZS90, Malvern Instruments Ltd., Shanghai, China). After sealing and storing the prepared DMY-AgNPs solution at 4 °C in a light-shielded environment, its stability was evaluated using a UV-vis spectrophotometer (350–800 nm) at 0, 45, and 90 d. The crystalline structure of DMY-AgNPs was investigated using powder XRD analysis (Ultima IV, Rigaku Corporation, Tokyo, Japan) from 0° to 90° with a voltage of 40 kV and a current of 30 mA. To characterize the synthesis mechanism of DMY-AgNPs, FTIR spectroscopy analysis (IRSpirit-T, Shimadzu Corporation, Shanghai, China) was carried out in the wavenumber range of 4000 to 400 cm^−1^.

### 3.4. Antibacterial Activity

Cultures such as MRSA ATCC 43300 and *S. aureus* ATCC 35218 were preserved in −80 °C as glycerol stocks. During the experiment, microorganisms were subcultured on LB plates and inoculated in LB broth. MIC, MBC, and a bacteriostatic ring of DMY-AgNPs were evaluated via microbroth two-fold dilution and the colony-forming units counting and punching method described in ref., respectively [52,53].

### 3.5. Toxicity Test

Fish maintenance and exposure to AgNPs adhered to the protocols outlined by Fernanda S. Dametto et al. [54], with all experiments conducted in accordance with the guidelines established by the Animal Care and Use Committee at the Institute of Hydrobiology, Chinese Academy of Sciences.

Healthy and synchronously hatched zebrafish embryos were selected at 12 h post fertilization (hpf) and exposed to varying concentrations of DMY-AgNPs in 12-well plates (20 embryo per well, with 4 mL of solution) until reaching 96 hpf. The DMY-AgNP solution was diluted using freshly prepared E3 medium without methylene blue (5 mM NaCl, 0.17 mM KCl, 0.33 mM CaCl_2_, 0.33 mM MgSO_4_, pH = 7.2–7.3). The control group was cultured with E3 medium. Observations of embryos development in each well were conducted using an ECLIPSE Ts2R inverted microscope (Nikon, Japan) at specific time intervals. Various endpoints, including heartbeat, hatching rate (Equation (1)), survival rate (Equation (2)), teratogenesis rate (Equation (3)), and body length, were assessed to evaluate the impact of DMY-AgNPs. Any embryo not displaying a heartbeat was promptly identified as deceased and removed from observation at each time interval [47].

(1) $$\mathrm{Hatching}\;\mathrm{rate}=\frac{\mathrm{Hatching}\;\mathrm{number}}{\mathrm{Total}\;\mathrm{number}\;\mathrm{of}\;\mathrm{embryos}}$$

(2) $$\mathrm{Survival}\;\mathrm{rate}=\frac{\mathrm{Survival}\;\mathrm{number}}{\mathrm{Total}\;\mathrm{number}\;\mathrm{of}\;\mathrm{embryos}}$$

(3) $$\mathrm{Teratogenesis}\;\mathrm{rate}=\frac{\mathrm{Teratogenesis}\;\mathrm{number}}{\mathrm{Total}\;\mathrm{number}\;\mathrm{of}\;\mathrm{embryos}}$$

### 3.6. MRSA-Infected Zebrafish and DMY-AgNPs Treatment

Adult zebrafish were exposed to MRSA via immersion following dermal abrasion, as described by Zhang, QH et al. [55]. Subsequently, they were fed a diet containing varying doses of AgNPs twice a day for a duration of 5 days. The fish feed was prepared according to the method described by Nathaniel J. Clark et al. [56]. Zebrafish were administered feed containing 0.0156, 0.0312, and 0.0625 μg of DMY-AgNPs per gram each time. Zebrafish would eat all the feed in less than one minute. Upon the completion of the treatment period, all fish were anesthetized by placing them on ice for 1–2 min. Subsequently, gills, livers, and intestines were harvested and subjected to histopathological examination [57].

### 3.7. Statistical Analysis

The data were expressed as mean ± standard deviation (SD). Statistical analysis was performed using the GraphPad Prism 8 (version 8.0.2 (263)), Origin 2024 (version 10.1.0.178) and Image J (1.8.0_345) [58]. The statistical significance was analyzed by one-way ANOVA (and nonparametric or mixed) using GraphPad Prism software (version 8.0.2 (263)). A statistical differences at *p*  <  0.05 was considered to be significant [59].

## 4. Conclusions

In summary, this study optimized the synthesis method of AgNPs using the efficient green reducing agent DMY. DMY, a natural dihydroxyflavonoid compound, serves as a stabilizing, reducing, and capping agent in AgNPs synthesis. Nanoparticle characterization was performed using techniques such as TEM, FTIR, and XRD. The results demonstrated that compared to the original method, DMY-coated AgNPs exhibited superior properties, including improved dispersity, good crystallinity, smaller particle sizes, and stability. Additionally, DMY-AgNPs displayed potent antibacterial effects against both standard and drug-resistant strains of *S. aureus*, and low toxicity to zebrafish embryos. In vivo infection and treatment experiments in MRSA-infected zebrafish indicated the favorable therapeutic effects of DMY-AgNPs on the gills, intestines, and livers. This study contributes to developing a better understanding of the impact of DMY-AgNPs on aquatic systems and provides recommendations for the safe use of DMY-AgNPs in aquaculture. Furthermore, in the future, the systemic toxicity and genetic effects of DMY-AgNPs on zebrafish should be evaluated. Simultaneously, the application of DMY-AgNPs in mammals, such as to anticancer and wound-healing uses, should be expanded.

## Data Availability

The authors do not have permission to share data. Or Data will be made available on request.
